# Predicting queue wait time probabilities for multi-scale computing

**DOI:** 10.1098/rsta.2018.0151

**Published:** 2019-02-18

**Authors:** Vytautas Jancauskas, Tomasz Piontek, Piotr Kopta, Bartosz Bosak

**Affiliations:** 1 Leibniz Supercomputing Centre of the Bavarian Academy of Sciences and Humanities, Boltzmannstraße 1, 85748 Garching near Munich, Germany; 2 Poznan Supercomputing and Networking Center, Institute of Bioorganic Chemistry of the Polish Academy of Sciences, ul Z. Noskowskiego 12/14, 61-704 Poznan, Poland

**Keywords:** multi-scale computing, machine learning, Naive Bayes

## Abstract

We describe a method for queue wait time prediction in supercomputing clusters. It was designed for use as a part of multi-criteria brokering mechanisms for resource selection in a multi-site High Performance Computing environment. The aim is to incorporate the time jobs stay queued in the scheduling system into the selection criteria. Our method can also be used by the end users to estimate the time to completion of their computing jobs. It uses historical data about the particular system to make predictions. It returns a list of probability estimates of the form (*t*_*i*_, *p*_*i*_), where *p*_*i*_ is the probability that the job will start before time *t*_*i*_. Times *t*_*i*_ can be chosen more or less freely when deploying the system. Compared to regression methods that only return a single number as a queue wait time estimate (usually without error bars) our prediction system provides more useful information. The probability estimates are calculated using the Bayes theorem with the naive assumption that the attributes describing the jobs are independent. They are further calibrated to make sure they are as accurate as possible, given available data. We describe our service and its REST API and the underlying methods in detail and provide empirical evidence in support of the method's efficacy.

This article is part of the theme issue ‘Multiscale modelling, simulation and computing: from the desktop to the exascale’.

## 1. Introduction

The issue of queue wait times comes up in many situations in High Performance Computing (HPC). We define queue wait time as the number of seconds between the time a job gets submitted to the resource manager (SLURM, PBS, LoadLeveller, etc.) and the time that the job starts executing. There is a trade-off here, in the sense that the more resources are requested, the faster the computing job will be finished, however, it will take more time for the resource manager to collect the required resources. There can be many situations in which the knowledge of the queue wait time is desirable. The users, for example, are interested in the queue wait time because it counts towards the total time between submission of a simulation or some other computing task and retrieving its results (time to completion). To the majority of users, the time spent waiting in the queue is no different from the time spent waiting until their simulation has finished executing. Therefore, it is of interest to the user to have an estimate of how long their job will spend queuing before submitting it. Having this information, they may tweak the amount of resources they request in order to get the results sooner. They may also consider submitting to another, less crowded, system. The issue of queue wait times can come up in scenarios where jobs are submitted by automated systems such as HPC middleware. It can also be beneficial in urgent computing [1–3] (when paired with preemption mechanisms). The proper selection of resources and assignment of tasks to them is particularly important in the multi-scale and multi-physics scenarios that come up very often in scientific simulations [4,5]. Since parts of the coupled multi-scale simulations may require for their efficient execution different types of computing resources to run on, in the ComPat project [6] we attempt to distribute parts of multi-scale jobs across heterogeneous resources [7]. To this end, we employed the QCG middleware, fully developed by Poznan Supercomputing and Networking Center [8]. QCG is a software stack that can virtually weld computing resources from different administrative domains into a single powerful supercomputing resource. This capability is essential for the ComPat applications to scale up towards exascale performance now, while there are only petascale systems available. The QCG middleware services control the whole process of execution of the multi-scale application on the production environment consisting of many Tier 0/1 computing resources in a way that satisfies users (job owners) and meets their application requirements as well as taking into consideration constraints and policies imposed by other stakeholders, i.e. resource owners. In the ComPat project, we put significant efforts into advancing the brokering capabilities of the QCG system. The new multi-criteria brokering plugin was implemented for the QCG-Broker service that allows selection of resources for applications based on multiple metrics, such as expected time to completion and expected energy usage. Thus a user, depending on their needs, can request the faster completion of a job or, as is currently innovative and essential for brokering of large computing jobs, the limitation of energy loss. In this scenario, the user specifies certain parameters that describe acceptable solutions and the brokering mechanism attempts to find an optimal, from the user point of view, set of resources that satisfies those parameters. The multi-criteria approach to resource selection and the practical use of queue wait time prediction needed to estimate the time to finish was the initial inspiration and the main motivation for the work described in this paper.

The general usage process of the ComPat system [7] is as follows:

(i) The Pattern Optimization Service, based on the knowledge of the application itself and the static information about the infrastructure, generates a list of assignment plans determining the search space for the optimal allocation of resources to computational kernels (parts of the multi-scale application). This list is submitted to the QCG-Broker service as a part of the job description with both the requirements for computing resources and definition of optimization criteria and limits.
(ii) Plans that are not realizable, e.g. due to unavailability of required resources, are removed from the list.
(iii) The specialized module of the QCG-Broker performs multi-criteria selection of the optimal feasible plan. In the process of selection, the three criteria, covering to the best of our knowledge the majority of real and potential scenarios, are taken into account:
  — Electricity needed to carry out calculations. (This criterion currently does not take into account other infrastructure-related costs such as cooling, for example.)
  — Time to completion—defined as the sum of the time waiting for resources and the time it takes to perform the simulation.
  — Time the computing infrastructure is in use—defined as the product of the number of cores and the hours of their use.
(iv) The multi-scale job is scheduled for execution and executed on selected resources with the help of QCG resource-level services.

Since the simultaneous optimization of many objectives for non-trivial cases is usually very complicated, it was decided to aggregate them through a single objective function, which is minimized. This function is determined as a weighted sum of many objective functions related to individual criteria, where the weights of individual criteria can be understood as a conversion of a given criterion into a common unit, e.g. monetary. The system allows for an optimal selection of resources in a scenario in which the total cost of commercial or scientific calculations depends on both the energy consumed and the use of resources, and at the same time penalty fees are charged for delays in exceeding the set date for the delivery of results.

As can be seen, among other requirements of such simulations are the constraints for the acceptable time to finish and, indirectly, queue wait time. In this work, we mostly focus on the theoretical and technical aspects related to the single time to finish criterion. In practice, the criterion and problem boil down to determining for each queue and given time the probability that the task with given requirements will be started in this queue at a given time. These parameters can be provided by the service described in this paper. The general interaction scenario between the ComPat system and the queue time prediction service described in this paper can be expressed in the following steps:

(i) A user or another service submits node description with the requirements for computing resources.
(ii) The system finds resources/sites/clusters that satisfy those requirements.
(iii) For each queue, we return information about the predicted queue wait times.
(iv) The user/service can decide which queue to choose in one of the following ways:
  — Via a specification of acceptable probability and time. That is, the user specifies that they require that their job starts within 1 h with a certainty of 90%. If no such queue exists, the job would not be submitted and an error will be returned.
  — By specifying acceptable time. Then the queue that can guarantee the highest certainty that the job will start before that time will be returned.
  — By specifying an acceptable probability, say 90%. The brokering algorithm will pick the option with the shortest queue wait time with that probability. The probability in this case represents how important is the queue wait time accuracy as a factor to the user. If the user specifies the probability as 0.5 (instead of 0.9), then they might get a shorter queue wait time, but the risk will also be higher that the prediction is not reliable.
  — Manually, having in mind all the information about the queue wait times and their probabilities.

There are several ways in which we could approach this problem. Batch schedulers often have functionality that allows them to estimate how much time a submitted job will stay in the queue. This estimate is adjusted as resources become available. However, the job has to be submitted first and estimates change with time. An additional disadvantage of this approach is that to get to know the waiting time the system has to start collecting resources, which results in costs on the resource provider side and can have impact on other estimations. Therefore, we cannot use this approach in the kind of scenarios we are interested in. We need a good estimate before submitting. We could use a regression approach, and for each queue, return an estimate for how long the job queue will be. There are various ways of doing this, starting from simply finding the most similar job in terms of Euclidean distance in attribute space to more elaborate symbolic regression methods. Without any kind of confidence analysis, this would present the user with a single number for each queue. It is not quite clear how a user should interpret those numbers. In particular, how much trust to put into them.

There is already literature available on the subject of tackling this problem. Smith [9–11] explores the use of execution and queue wait time predictions in order to select resources for executing applications on computational grids. The author relies on application run time predictions in order to perform a simulation of the scheduler (in their case, it is a simple ‘first come, first served’ scheduler). This is not suitable for our needs. Getting an accurate estimate of program execution times would be really difficult across different systems with many users. Furthermore, the schedulers are different and implement different scheduling strategies. Some of them are quite complex (or impossible) to accurately simulate (e.g. fair-share scheduling). Li *et al.* [11] explore queue wait time prediction in a similar context as in our paper. They take a similar approach to [10] and simulate a specific scheduler using job run time predictions. However, as they assume the Maui scheduler [12] which is not widely used anymore, and as they base their work on simulations of the scheduler, their work cannot be applied in our case.

Gao *et al.* [13] use a genetic algorithm to optimize job scheduling, however their approach does not apply to us due to the fact that we need to estimate job queue wait time probabilities. Chen *et al.* [14] use Naive Bayes, but in a different context, namely optimizing MapReduce partitioner.

In this paper, we propose a novel method based on the well-known technique of Naive Bayes classification. The time is discretized to a certain granularity that is chosen when deploying the system. We divide time in pairs of intervals and solve the problem of assigning jobs to two classes—whether the job will start before time *t*_*i*_ or whether it will start after. We use the Naive Bayes formula (Bayes theorem with the assumption that attributes are independent of each other) and probability calibration techniques to arrive at a probability for each of the two events. The output of the method is a list of tuples of the form (*t*_*i*_, *p*_*i*_) where *t*_*i*_ is time in seconds and *p*_*i*_ is the probability that the job will start before time *t*_*i*_. The user then has a set of probabilities to manually examine or they can let the brokering algorithm choose the resources for them.

In §2, we describe the method itself. This includes the data collection techniques we have employed, numeric attribute discretization and probability calibration. Each of these are important to the performance of our method. We then provide empirical evidence about the quality of the performance of our method in §3 and finally conclude our findings in §4.

## 2. Naive Bayes for queue wait time prediction

In this section, we will explain the proposed method. The whole process is divided into three main subtasks—data collection, model building and the actual prediction service. We start with an overview of the aforementioned data processing pipeline.

### (a) Method outline

The method we have chosen to use consists of several consecutive stages. We outline them in the list below. This is not a completely straightforward implementation of the Naive Bayes method. First of all, we have numerical attributes (they are integers, but the range is too broad to treat them as categorical). Therefore either discretization, density function fitting or density function estimation has to be used to get the probabilities required to apply the Bayes formula. We have decided to use discretization since it is not exactly clear what the probability density functions are for these attributes and using kernel density estimation gives poor results in comparison. Since simple binning did not prove satisfactory, we employ a more sophisticated method that works by assigning bin boundaries in order to maximize information about the class. The exact method used is described in the §2c. We then use several Naive Bayes classifiers—one for each queue wait time point. For example, if the user specifies 4 points—10 min, 1 h, 6 h and 24 h—there will be four different classifiers. The first one to decide whether the job will queue for less than 10 min or not, the second one to decide whether the job will queue for less than 1 h or not, and so on.

The probabilities produced by Naive Bayes classifiers are known to be inaccurate (e.g. [15,16]). This will be discussed in §2e. The probabilities can be calibrated but methods for doing this generally only work for two class problems. Therefore, a multi-class problem such as ours needs to be converted into several two-class problems. There are several known ways for doing this [17]. The one we have chosen is a simple binning-based approach that will be discussed in §2c.

Our method consists of the following stages:

(i) Collect data and store it. This step is continuously ongoing. Discussed in more detail in §2b.
(ii) Build models from that data (this step is triggered on demand, for example, daily):
  — Discretize numerical attributes. Discussed in more detail in §2c.
  — For each time boundary, construct and store the corresponding Naive Bayes model. Discussed in more detail in §2d.
  — Calculate the information that will be needed to calibrate the probabilities. Discussed in more detail in §2e.
(iii) Predict queue wait times for incoming jobs:
  — For each time boundary calculate the probability that the job will start executing before that time boundary and the probability that it will start after.
  — Calibrate the probabilities for more accurate results.
  — Return the probability data to the person or service that requested it.

Let us consider these stages in more technical detail. Most of the methods here are well known in the machine learning community. However, this exact combination was determined by us to have the best performance for our problem.

### (b) Data collection and analysis

Queue wait time will likely depend on many factors. Primarily, it is the result of the system state at the moment (that is how much of the resources are taken versus what resources are available on the system) and the amount of resources requested for the specific job. The more resources the user requests, the more time it will take to allocate them. We tried to decide which specific factors will influence this. These can be grouped as follows:

— The amount of resources requested. Usually, the more resources are requested in order to execute a job the more time it takes for the resource manager to secure those resources.
— The state of the system. This will usually boil down to how much of the resources are currently taken by other jobs. Usually, this will be the resources available to a particular queue. In general, we should suppose that the more resources are currently in use, the longer it will take for the scheduler to gather the requested resources.
— Historic use of the system for particular users. Generally, it can be assumed that the more a particular user used the system in the near past the harder it will be for them to get resources. This, of course, is only true if the resource manager tries to implement some sort of fairness in the resource management process.
— The future state of the system. That is when the currently running jobs finish, how many new jobs are submitted and with what priority, etc. We will not take these factors into consideration because it is out of scope of the current work.

We collect data from all the sites participating in the ComPat project. Data collection is done by parsing the output of various scheduler tools. The tools and their output will depend on the resource manager (e.g. SLURM, LoadLeveler, PBS) used on that system. Therefore for each scheduler we had to implement a separate data gathering script. They all monitor the system until a new job arrives and after it is finished stores its queue and execution time as well as other attributes in the database.

The attributes that describe the jobs and upon which the prediction as to the queue wait time is made are very important. They have to contain enough information about what the queue wait time can be expected to be. It is not, in general, possible to know which attributes are the most descriptive before collecting them. Therefore, we collect information about a lot of different aspects of each job. See the list below for information about which attributes we store.

(i) **(1–8)** Job attributes
  (a) site (string)—the name of the site to which the job was submitted
  (b) username (string)—name of the user
  (c) class (string)—queue/class/partition name
  (d) when_submitted (integer)—UNIX timestamp for when the job was submitted
  (e) time_requested (integer)—how much wallclock time the user has requested
  (f) cpu_time_requested (integer)—how much cpu time the user has requested
  (g) tasks_requested (integer)—how many tasks (cpus) the user has requested
  (h) nodes_requested (integer)—how many nodes the user has requested
(ii) **(9–20)** System attributes—describe the state of the whole cluster
  (a) sys_jobs_running (integer)—how many jobs are currently running
  (b) sys_jobs_queued (integer)—how many jobs are currently queued for execution
  (c) sys_tasks_running (integer)—how many tasks are currently running (how many cpus are currently used)
  (d) sys_tasks_queued (integer)—how many tasks are currently queued
  (e) sys_nodes_running (integer)—how many nodes are currently running
  (f) sys_nodes_queued (integer)—how many nodes are currently queued
  (g) sys_time_running (integer)—the sum of wallclock time requested by the jobs already running
  (h) sys_time_used (integer)—the sum of time already consumed by running tasks (the sum of time the tasks are already running for)
  (i) sys_time_queued (integer)—the sum of wallclock time requested by the jobs that are currently waiting for execution
  (j) sys_cpu_time_running (integer)—the sum of CPU time requested by the jobs that are currently running
  (k) sys_cpu_time_used (integer)—the sum of CPU time already used up by the running jobs
  (l) sys_cpu_time_queued (integer)—how much CPU time is currently queued
(iii) **(21–33)** Class attributes—describes the state of the queue or partition. These are the same as system state variables but with class prefix instead of sys prefix. The meaning of attributes is analogous. For example, class_tasks_running means how many tasks are currently running in that queue.
(iv) **(34–46)** User attributes—describes the state of the user (information about the user's jobs). Again, identical to sys and class attributes in meaning, but describes jobs currently running, queued, etc., for particular users. The prefix is user instead of sys or class. For example, user_tasks_running.

### (c) Discretizing numerical values

There is an additional complication to using Naive Bayes for our purposes. It is very easy to calculate the required probabilities if the attributes are nominal (consisting of a small set of distinct values). Our attributes are generally numeric. While they are integers the ranges are so large that they cannot in practice be treated as nominal. There are two main ways one can approach this.

(i) Try to estimate the probability distribution. This can be done by either fitting a probability distribution to data (provided we have a reasonable assumption about what that probability distribution is) or using kernel density estimation techniques.
(ii) Discretize numerical attributes, turning them into nominal attributes. The values are then binned into a certain number of bins. Then the numerical attributes are changed to nominal attributes by replacing the attribute values by the bin ids that the attribute falls in.

The second approach (discretization) worked much better in our preliminary tests, therefore that is what we decided to use.

The method we chose for discretizing numerical values is that of Fayaad & Irani [17]. It is a fairly sophisticated method intended to maximize information about which class the decision vector belongs to when assigning bin boundaries. This is in contrast to simple, unsupervised discretization methods, which proved to perform poorly during the development of our method.

### (d) Naive Bayes classifiers

We have chosen to use a Naive Bayes classifier approach to solve our problem. The Naive Bayes classifier is a simple machine learning technique based around the Bayes theorem. It has been studied extensively since the 1950s. Empirical studies of the technique were performed by Rish *et al.* [16], Lewis [18] and many others. Rennie *et al.* [15] performed a study of the assumptions employed by the Naive Bayes classifiers. There are several reasons for choosing it in our case. First of all, instead of just reporting the class that the classifier predicts an object belongs to, it returns probabilities of an object belonging to each class. This is exactly what we need. The probability is that the Naive Bayes classifiers reports will not be accurate because of the independence assumption that the classifiers rely on (the assumption that the attributes are mutually independent), however we can correct this using a simple calibration procedure.

The process of predicting queue wait time probabilities boils down to determining what to put in the Bayes formula:

2.1 $$p(c|E)=\frac{p(E|c)p(c)}{p(E)}.$$

Here, the probability *p*(*c*|*E*) is the probability of an object *E* belonging to class *c*. Probability *p*(*E*|*c*) is the probability that if an object is of class *c* it is the object *E*. Probability *p*(*c*) is the probability of any object of belonging to class *c*. Probability *p*(*E*) is the probability of an object being the object *E*. The value of *p*(*c*) is easy to accurately estimate provided there is enough training data. In our case, the supply of training data is not limited in any sense. We can simply count the number of instances of a given class in the training set and divide by the number of samples.

The most complicated part is calculating this conditional probability:

2.2 $$p(E|c)=p(x_{1},x_{2},\ldots ,x_{n}|c).$$

Here *x*_*i*_ is the event that attribute *i* takes on a value *x*_*i*_. Accurately estimating *p*(*E*|*c*) requires knowing the exact dependencies between variables (their joint probability distribution). However, a simpler approach that often works in practice is to just assume that the attributes are independent. In that case to get the probability we multiply the conditional probabilities of each attribute (given that the object belongs to class *c*) together. Values *p*(*x*_*i*_|*c*) can be calculated from the training data by counting the relative frequencies of attribute *i* taking on value *x*_*i*_.

2.3 $$p(E|c)=\prod_{i=1}^{n}p(x_{i}|c).$$

We are left with one part of the equation and that is *p*(*E*). Since it does not depend on the class it is merely a scaling factor. Instead of trying to calculate it, we can ignore it altogether. Then the output of the formula would not be a probability. However, since we know that the values of *p*(*t* < *t*_*i*_|*E*) and *p*(*t*≥*t*_*i*_|*E*) have to add up to one we can simply scale them afterwards by dividing each of them by their sum. Here, *t* is the time the job spent queuing and *t*_*i*_ is one of the time boundaries.

Let us now suppose that we want to predict queue wait time using 1 h intervals. If we want to limit prediction to, say, 72 h we will have the following time boundaries: *t*_1_ = 0, *t*_2_ = 3600, *t*_3_ = 7200, …, *t*_72_ = 259, 200 and the following models: *p*(*t* < *t*_1_|*E*), *p*(*t* < *t*_2_|*E*), *p*(*t* < *t*_3_|*E*), …, *p*(*t* < *t*_72_|*E*). Now when given an attribute vector *E* we will calculate the output of each such model. There will be 72 different outputs representing 72 probabilities.

### (e) Calibrating probabilities

It is well known that the probabilities of Naive Bayes classifiers tend to be skewed. Generally (in practice), they will be squashed either towards one or zero depending on whether the decision vector belongs to the given class or not, see [19]. This is not a problem when using them strictly for classification. That is because if we have two decision vectors ***x*** and ***y*** and *s*(***x***, *c*) and *s*(***y***, *c*) are the probabilities given by the Naive Bayes Classifier that the vectors belong to class *c*, then from *s*(***x***, *c*) < *s*(***y***, *c*) it follows that *p*(*c*|***x***) < *p*(*c*|***y***), see [19]. The reason why the probabilities given by the Naive Bayes Classifier are different from actual probabilities is the naive assumption employed by the classifier. In general, this assumption is false as there will be correlations between attributes. However, the real values of the probabilities are not important when using it simply as a classifier, as long as the above inequalities and the relationship of logical implication between them holds. This is further investigated and more rigorously shown by Domingo & Pazzani [19]. We are interested in the actual probabilities as opposed to classifier scores, because it is based on the actual probabilities that the user will want to make the decision. In particular, they will rely on what is to them an acceptable probability that a job will start before a specific time.

It is possible to calibrate the probabilities and thus increase their accuracy. Several ways of doing this were proposed in the literature. One of the first simple and practically useful methods was published by Platt [20]. It is meant to transform the outputs of Support Vector Machines (SVMs) into posterior probabilities that a decision vector belongs to a certain class. The method assumes binary classification problems and works by sorting the distances between the support vector and a decision vector in increasing order and fitting a sigmoid function through it. This function is then used to translate SVM outputs to probabilities. Further work in this area was done by Zadrozny & Elkan [21,22], who examined the use of probability calibration for SVMs, decision trees and Bayes classifiers. They also proposed a new method based on isotonic regression. For Naive Bayes, they proposed a method based on simple binning of vectors sorted by their Naive Bayes score. This is the method we chose to use for our problem. It is simple to implement and we have a considerable amount of data which makes binning feasible.

For the benefit of the reader, we provide a short explanation of the method here. Note that we are dealing with a two-class problem. The classes are: ‘the job will start executing sooner than *t*_*i*_ amount of time’ and ‘the job will start later than *t*_*i*_’. The value *t*_*i*_ is the *i*th time point. If we have decided, for example, to calculate the probabilities with the granularity of 1 h then *t*_1_ will be—the job will start within an hour, *t*_2_ will be—the job will start within 2 h and so on. For each *t*_*i*_, we calculate the Naive Bayes probability using the method described in §2d. This is done for each job in the training set. We then sort all the jobs according to this value. In the end, there will as many such sorted sequences of jobs as there are time points.

Then we bin the sorted jobs using equal frequency binning. That is, the number of records in each bin has to be the same. If that is not exactly possible (the number of records does not divide by the number of bins), it has to be as equal as possible. The number of bins will define the coarseness of the adjusted probability estimates. The fewer bins, the fewer discrete values probabilities can take. There are several considerations when choosing the number of bins. More bins means higher accuracy. However, it also means there needs to be enough training data to fill those bins. Bin boundary values are then the uncalibrated Naive Bayes probabilities of the first and last job in the bin.

The calibration itself is simple. For each probability to be calibrated, we calculate the bin it falls in. The bin boundaries were calculated in advance using the method we described in the previous paragraph. Once we have the bin we calculate the class ratio for that bin. The class ratio is the number of jobs in the training data in that bin that belong to the before class versus the after class. This ratio is the calibrated probability.

## 3. Results

In this section, we will provide some empirical proof of the efficacy of our method. Two experiments were performed, each examining different aspect of the accuracy of the predictions performed by the service. We have chosen one of the queues for which data were collected during the course of the ComPat project [6]. It was chosen on the basis of being heavily used and therefore having a relatively long average queue wait time. Underused systems are unlikely to be interesting in terms of queue wait time predictions. Almost 1 year's worth of data was used for the experiment, starting from 23 August 2017 and continuing until 26 April 2018. The data consists of information about 220 185 jobs submitted during that time period to the Linux Cluster (CooLMUC-2) at Leibniz Supercomputing Centre of Bavarian Academy of Sciences. The cluster is quite actively used and uses SLURM as its scheduler. The average queue wait time for this system is roughly 15 h. As such we believe it is a good case to study.

### (a) Comparing discretization methods

Here we compare the methods for estimating the probability distributions of job attributes. These are required to calculate the joint probability distribution which is then used with the Bayes formula to produce a probability estimate. The results are given in the table. We give the percentage of correctly classified instances for several selected time points for each of the methods. First the attribute values were discretized with the given method, then the Naive Bayes classifier was used. This uses the 220 185 job data mentioned previously. As can be seen the method we have used outperforms the others in term of classification accuracy. The other method is simple binning into equal width bins with optimal bin number estimation, equal frequency binning (bins contain approximately the same number of instances and kernel density estimation). For exact details of how these are implemented see [23].

**Table:** 

time point	Fayyad92 (%)	binning (width) (%)	binning (frequency) (%)	KDE (%)
1 m	90.2277	71.8309	88.2643	77.8277
30 m	88.3553	60.614	75.3694	78.7299
1 h	89.4139	69.8588	75.441	80.0308
4 h	92.8672	71.3324	78.5722	82.9839
8 h	94.1034	75.2394	78.7376	82.1537
16 h	94.5787	79.7136	78.729	85.8201

### (b) Prediction accuracy

One of the aspects by which the proposed service can be evaluated is the accuracy of the binary predictions that it makes. As was stated before, for each time division point, it returns the probability for the event that the job will start before that point. If that probability is higher than 0.5, we have a reason to believe it will, otherwise we have a reason to believe it would not. Here we measure how often this prediction is correct. We treat it as a binary classification problem at each time point. We have assumed 15 such points and hence 15 problems. For each, we measure the ratio of incorrect guesses to the number of all jobs in the dataset and plot the results.

We perform the experiment for prediction accuracy using 10-fold cross-validation [24]. It works as follows:

(i) Divide time using 60 × 2^*i*^ where *i*∈[0, 15] seconds as division points. This results in the division points of 60, 120, 240, 480 and so on seconds up to 983 040 s. These time points are the same ones as used by the service in operation. The logic behind choosing exponentially increasing time points is that the service likely becomes less accurate with longer queue times because of factors like newly submitted jobs, jobs finishing, etc. Therefore, it does not make sense to use fine-grained time points for longer time periods.
(ii) Divide data at random into 10 equally sized parts.
(iii) For each part build a model using the remaining nine parts. Measure prediction accuracy on that part by comparing model predictions to actual data. A job will be considered misclassified if the probability is higher for the wrong class. The two classes being that a job will start before and after the given time point.
(iv) For each of the 15 time points, plot the number of jobs that were misclassified, jobs that were correctly classified, etc.

**Figure 1.**
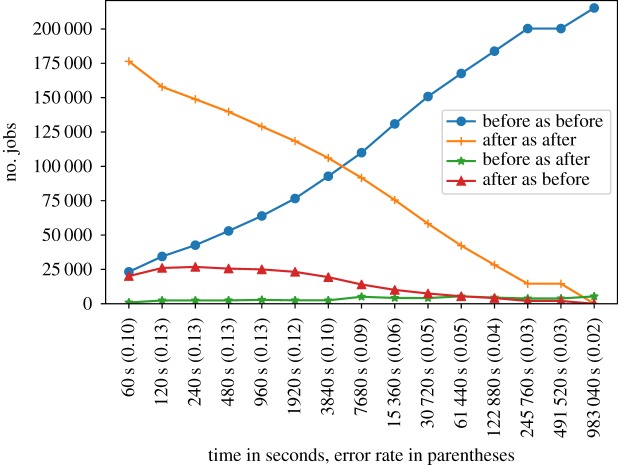
Prediction accuracy plot. It shows the numbers of correctly and incorrectly classified jobs for each time point. The *x*-axis shows the time points in seconds. The *y*-axis shows the number of jobs that were correctly and incorrectly classified. There are four cases—jobs correctly classified as starting before the time point, jobs correctly classified as starting after the time point, jobs incorrectly classified as starting before the time point and jobs incorrectly classified as starting after the time point. (Online version in colour.)

We can see the results in figure 1. The *y*-axis is linear and the *x*-axis is logarithmic (each time point is double that of the previous time). Job counts are displayed in the *y*-axis and the time points are in the *x*-axis. As can be seen from the plot, there is a slight tendency to misclassify jobs as starting before the given time point. The exact reason for this is unclear. The error rate (the ratio between the number of incorrectly classified instances and the number of all instances) is shown in parentheses in the *x*-axis labels. Since this is a queue with a long average queue wait time, a lack of accuracy at lower wait times is not surprising. However, it can be seen that the proposed method is relatively accurate—resulting in below 10% incorrectly classified instances in most cases, going down to around 5 for cases close to the average queue wait time for this data set.

**Figure 2.**
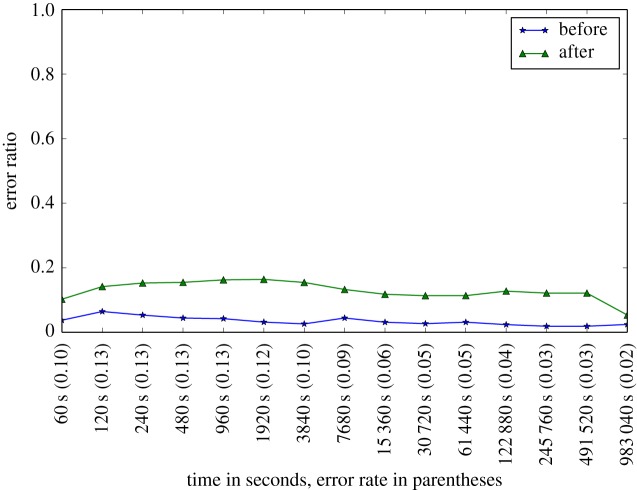
Prediction error rate plot. It shows the error ratio for each time point. The *x*-axis shows the time points in seconds. The *y*-axis shows the ratio between incorrectly classified instances of each class versus the total number of instances in that class. (Online version in colour.)

In figure 2, we provide a plot of error rates for each of the time points examined.

### (c) Probability accuracy

It is important to define what we call the probability that a job will start within *t* seconds. A simple intuitive definition should satisfy at least the following criterion: if the probability is reported as *x*, then we expect approximately *x* × 100% of the jobs with that reported probability to start within *t* seconds as the number of jobs in the testing set increases. For example, if we have 100 jobs that were all given a probability of 0.2 to start within 10 min we expect that around 20 of them will start within 10 min.

**Figure 3.**
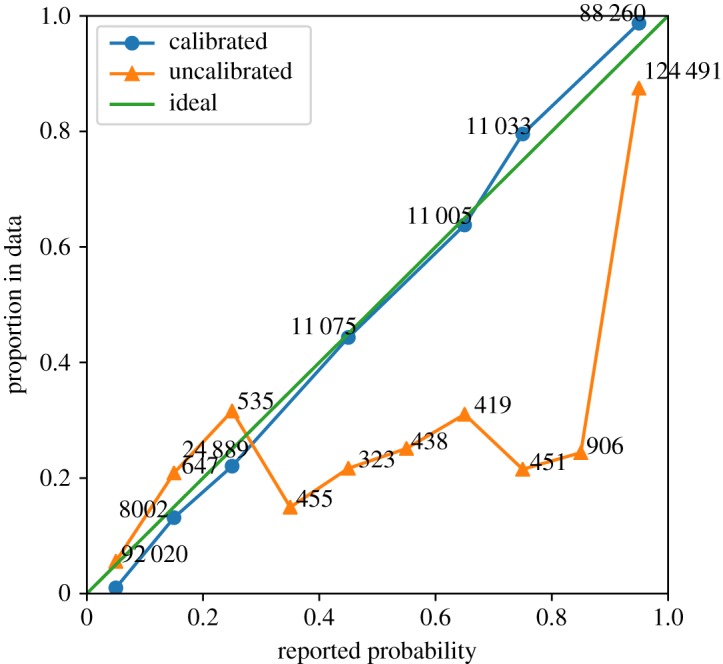
Probability calibration plot for a SLURM-based cluster at the time point of 2 h. Ideally, the calibrated line should fall exactly on the *y* = *x* diagonal. The numbers next to the dots signify the number of samples in that particular bin. (Online version in colour.)

In figure 3, we see a plot based on the same data as for the previous experiment. In this case, we have chosen only one of the time points, namely 7680 s or around 2 h. The *x*-axis shows the reported probability and the *y*-axis shows the actual proportion in the testing data. As can be seen the calibrated probabilities are close to the ideal which is the line *y* = *x*, while the uncalibrated probabilities are skewed. The numbers on the plot points signify the number of jobs in that bin.

## 4. Conclusion

In this paper, we have described a method for calculating accurate probabilities for discrete queue wait times. The method allows us to give the client a probability of the job starting before a specified time. We have also developed a service that reports these probabilities for a user-specified set of times. Therefore, the user can choose the confidence level they are comfortable with when submitting a job. Several computing resources can then be compared in this regard. For example, if it is important to the user that a job starts running as soon as possible they will submit a request for resources at a site that gives a high probability of a low time. If it is not very important the user may accept a riskier option. This is then used as a basis for a brokering mechanism for multi-scale computing problems. In other words queue wait time probabilities are incorporated into the decision of where to submit portions of multi-scale jobs. Parts of multi-scale simulations will have different requirements for optimal execution and queue wait times may be a part of them.

The proposed method is based on the Naive Bayes formula and the probabilities are further calibrated to ensure greater accuracy. The results show that the reported probabilities correspond well to the intuitive notion of a probability for a queue wait time. That is, if we have a certain number of jobs and for each of them the assigned queue wait time probability is, for example, 0.3, we expect 30% of them to start within the given time period. The same is true for other probabilities. Evaluating a large sample of jobs on the LRZ Linux Cluster, we provide empirical evidence that this is indeed the case when using the proposed approach.

A web service API is provided that was then used by the brokering system to make automated decisions as to where to submit parts of multi-scale simulations taking into account queue wait time probabilities. This API is very general and can be used by brokering systems and for other purposes. The service is being used by the brokering mechanism in the ComPat project. It runs on a Virtual Machine and communicates via the API we have developed. We hope that with further work it can provide an accurate and useful method to incorporate queue wait times into the multi-scale brokering decision making.

VJ was responsible for finding the combination of methods that give practically usable results when using queue wait time prediction in multi-scale computing scenarios. He was also responsible for the software implementation of the queue wait time prediction service.

TP, BB, PK were co-responsible for developing the concept of the work including defining the functional requirements of the service and programming interfaces. In addition, they participated as consultants in the field of HPC systems and were responsible for the integration and use of the described system with the QCG-Broker service.

## Data accessibility

This article has no additional data.
